# Overview and Methodology of the Adolescent Behaviors and Experiences Survey — United States, January–June 2021

**DOI:** 10.15585/mmwr.su7103a1

**Published:** 2022-04-01

**Authors:** Adriana Rico, Nancy D. Brener, Jemekia Thornton, Jonetta J. Mpofu, William A. Harris, Alice M. Roberts, Greta Kilmer, David Chyen, Lisa Whittle, Michelle Leon-Nguyen, Connie Lim, Andrew Saba, Leah N. Bryan, Jennifer Smith-Grant, J. Michael Underwood

**Affiliations:** ^1^Division of Adolescent and School Health, National Center for HIV, Viral Hepatitis, STD, and TB Prevention, CDC; ^2^ICF International, Rockville, Maryland

## Abstract

Many U.S. schools closed nationwide in March 2020 to prevent the spread of COVID-19. School closures and online-only instruction have negatively affected certain students, with studies showing adverse effects of the pandemic on mental health. However, little is known about other experiences such as economic and food insecurity and abuse by a parent, as well as risk behaviors such as alcohol and drug use among youths across the United States during the pandemic. To address this gap, CDC developed the one-time, online Adolescent Behaviors and Experiences Survey (ABES), which was conducted during January–June 2021 to assess student behaviors and experiences during the COVID-19 pandemic among high school students, including unintentional injury, violence, tobacco product use, sexual behaviors, and dietary behaviors. This overview report of the ABES *MMWR Supplement* describes the ABES methodology, including the student questionnaire and administration, sampling, data collection, weighting, and analysis.

ABES used a stratified, three-stage cluster probability-based sampling approach to obtain a nationally representative sample of students in grades 9–12 attending public and private schools. Teachers of selected classes provided students with access to the anonymous online survey while following local consent procedures. Data were collected using a 110-item questionnaire during January–June 2021 in 128 schools. A total of 7,998 students submitted surveys, and 7,705 of these surveys had valid data (i.e., ≥20 questions answered). The school response rate was 38%, the student response rate was 48%, and the overall response rate was 18%. Information on mode of instruction and school-provided equipment was also collected from all sampled schools.

This overview report provides student- and school-level characteristics obtained from descriptive analyses, and the other reports in the ABES *MMWR Supplement* include information on substance use, mental health and suicidality, perceived racism, and disruptions to student life among high school students. Findings from ABES during the COVID-19 pandemic can help guide parents, teachers, school administrators, community leaders, clinicians, and public health officials in decision-making for student support and school health programs.

## Introduction

Beginning March 2020, many U.S. schools closed nationwide to prevent transmission of SARS-CoV-2, the virus that causes COVID-19 (*1*). Although studies indicate that COVID-19 illness is generally less severe in children than adults (*2*), children are still at risk for developing severe illness and complications from COVID-19. In addition, the pandemic has had negative effects on adolescents’ mental health (*3*). Compared with 2019, the proportion of mental health–related emergency department visits in 2020 increased approximately 31% among youths aged 12–17 years (*3*). On January 13, 2021, approximately 50% of all students in the United States were receiving online-only instruction (*4*), and research has shown that online-only instruction has had a negative effect on the mental health of adolescents (*5*). Although these studies provide data on the effects of the COVID-19 pandemic on mental health among youths, little is known about other experiences such as economic and food insecurity, abuse by a parent, and other risk behaviors youths might have engaged in during this time, such as alcohol and drug use. To assess youth behaviors and experiences during the COVID-19 pandemic, CDC developed and conducted the Adolescent Behaviors and Experiences Survey (ABES) among students in grades 9–12. 

In spring 2020, CDC used funds from the Coronavirus Aid, Relief, and Economic Security (CARES) Act to launch ABES (https://www.govinfo.gov/app/details/PLAW-116publ136). CDC subject matter experts from the Division of Adolescent and School Health program, in collaboration with CDC’s COVID-19 Response Team, rapidly developed and implemented ABES by adapting methodology from the national Youth Risk Behavior Survey (YRBS), part of the nation’s largest surveillance system dedicated to adolescent health (*6*). ABES asked students about six categories of behaviors included in a typical YRBS questionnaire: 1) behaviors that contribute to unintentional injury and violence, 2) tobacco product use, 3) alcohol and other drug use, 4) sexual behaviors, 5) dietary behaviors, and 6) physical inactivity. In addition to these questions, ABES included one question about students’ perceptions of racism and 12 questions about the following experiences during the COVID-19 pandemic: 1) increased use of alcohol and drugs, 2) economic and food insecurity, 3) abuse by a parent, 4) poor mental health, 5) school and social engagement, and 6) use of telemedicine. This overview report describes the 2021 ABES methodology, including the online questionnaire and administration, sampling, data collection, weighting, and descriptive analyses. In addition, this *MMWR Supplement* also includes reports on substance use, mental health and suicidality, perceived racism, and disruptions to student life among high school students. Findings from ABES during the COVID-19 pandemic can help guide parents, teachers, school administrators, community leaders, clinicians, and public health officials in decision-making for student support and school health programs.

## ABES Methodology

### Overview

ABES was a one-time, online survey conducted during January–June 2021 to assess student behaviors and experiences during the COVID-19 pandemic. ABES surveyed high school students in grades 9–12 attending U.S. public and private schools. Classes were randomly selected to participate within a nationally representative sample of schools. Because of the different instructional models used across the nation during the pandemic (i.e., in-person only, virtual only, and hybrid), ABES was designed as a self-administered, anonymous survey that was administered online. The online administration allowed each school and teacher the flexibility to decide whether students completed the survey during instructional time or on their own time. In addition to student-level data, school-level data (e.g., instructional mode and school-provided equipment) also were collected.

### School Characteristics

The COVID-19 pandemic resulted in modifications of the learning environment, and many students faced several challenges, including lack of adequate resources at home (*7*). Therefore, collecting information on school characteristics during the fielding of ABES was important. School staff reported the school’s instructional model (in-person only, virtual only, or hybrid) at the time of recruitment; hybrid schools had a portion of a school’s student body receiving in-person instruction while others received virtual instruction. School staff members also reported whether the school had reduced class sizes and whether they provided laptops or Chromebooks, tablet computers, and Wi-Fi hotspots to students for home use.

### Student Online Questionnaire and Administration

Students completed ABES questionnaires in English or Spanish using a secure URL on any Internet-connected device. They were required to watch a 2-minute video (with an option of an English version, with English audio, or a Spanish version, with English audio and Spanish subtitles) and read brief instructions before starting the questionnaire. On average, students completed the 110-item questionnaire in 30 minutes. Although students were encouraged to finish the survey in one sitting, they were able to save their answers, stop the survey, and resume the survey as needed. Cumulative timing data were captured for the entire time a student was logged in, thus a meaningful range of completion time is not available. The survey included 97 questions from the 2021 national YRBS questionnaire. Six of these questions were modified to allow students who were attending school only virtually to indicate that a question asking about a behavior on school property did not apply. The questionnaire also included 12 new questions (not included on the YRBS questionnaire) assessing COVID-19–related behaviors and experiences and one new question on perceived racism (Box). The questionnaire was designed with no skip patterns; however, students could skip questions they did not want to answer. (Questionnaires in English and Spanish are available at https://www.cdc.gov/healthyyouth/data/abes.htm.)

BOX **Student survey questions on COVID-19 and perceived racism — Adolescent Behaviors and Experiences Survey, United States, January–June 2021**

**COVID-19 questions**

Do you agree or disagree that you drank more alcohol during the COVID-19 pandemic than before it started?

- Strongly agree
- Agree
- Not sure
- Disagree
- Strongly disagree

Do you agree or disagree that you used drugs more during the COVID-19 pandemic than before it started? (Count using marijuana, synthetic marijuana, cocaine, prescription pain medicine without a doctor’s prescription, and other illegal drugs.)

- Strongly agree
- Agree
- Not sure
- Disagree
- Strongly disagree

During the COVID-19 pandemic, did a parent or other adult in your home lose their job even for a short amount of time?

- My parents and other adults in my home did not have jobs before the COVID-19 pandemic started.
- Yes
- No

During the COVID-19 pandemic, did you lose your paying job even for a short amount of time?

- I did not have a paying job before the COVID-19 pandemic started.
- Yes
- No

During the COVID-19 pandemic, how often did you go hungry because there was not enough food in your home?

- Never
- Rarely
- Sometimes
- Most of the time
- Always

During the COVID-19 pandemic, how often did a parent or other adult in your home swear at you, insult you, or put you down?

- Never
- Rarely
- Sometimes
- Most of the time
- Always

During the COVID-19 pandemic, how often did a parent or other adult in your home hit, beat, kick, or physically hurt you in any way?

- Never
- Rarely
- Sometimes
- Most of the time
- Always

Do you agree or disagree that doing your schoolwork was more difficult during the COVID-19 pandemic than before the pandemic started?

- Strongly agree
- Agree
- Not sure
- Disagree
- Strongly disagree

During the COVID-19 pandemic, how often was your mental health not good? (Poor mental health includes stress, anxiety, and depression.)

- Never
- Rarely
- Sometimes
- Most of the time
- Always

During the COVID-19 pandemic, did you get mental health care, including treatment or counseling for your use of alcohol or drugs, using a computer, phone, or other device (also called telemedicine)?

- Yes
- No

During the COVID-19 pandemic, how often were you able to spend time with family, friends, or other groups, such as clubs or religious groups, by using a computer, phone, or other device? (Do not count attending school online.)

- Never
- Rarely
- Sometimes
- Most of the time
- Always

During the COVID-19 pandemic, did you get medical care from a doctor or nurse using a computer, phone, or other device (also called telemedicine)?

- Yes
- No

**Perceived racism**

During your life, how often have you felt that you were treated badly or unfairly in school because of your race or ethnicity?

- Never
- Rarely
- Sometimes
- Most of the time
- Always

In participating schools, after parental permission was granted and a student agreed to participate, teachers of selected classes provided students with instructions for accessing the survey and a randomly generated login code that allowed for completely anonymous participation. Among eligible students, 2.6% had a parent who refused to allow participation, and an additional 4.3% did not return a permission form when active consent was required. To help maintain privacy, only one question appeared on the screen at a time. Images were added for questions related to use of tobacco products, substances, and contraceptives to improve respondents’ recognition and understanding of the questions. 

### Sampling

ABES used the same sampling methods as the national YRBS, except that a larger sample was drawn in anticipation of lower response rates (*6*). The ABES sampling frame included public and private schools with grades 9–12 in all 50 U.S. states and the District of Columbia. Combined data obtained from MDR (formerly Market Data Retrieval) and the National Center for Education Statistics (NCES) were used to create the sampling frame. Public schools were identified from the Common Core of Data (https://nces.ed.gov/ccd), and private schools were identified from the Private School Survey (https://nces.ed.gov/surveys/pss), both of which are NCES databases. The sampling frame excluded alternative, special education, U.S. Department of Defense, Bureau of Indian Education, and vocational schools that serve students who are also enrolled in another public school. Home-schooled students and students who dropped out of high school were not eligible for ABES if they were not enrolled in a school. 

ABES used a stratified, three-stage cluster sampling approach to obtain a nationally representative sample of students. The first sampling stage consisted of primary sampling units (PSUs) encompassing a county, portion of a county, or a group of counties. A total of 81 PSUs were selected across 16 primary strata by urban and nonurban location and percentage of non-Hispanic Black and Hispanic or Latino (Hispanic) students in the PSU. The second sampling stage consisted of 335 secondary sampling units (SSUs), defined as physical schools with grades 9–12 or linked schools combined to provide the four grades. At the first and second stages, the sample was selected with probability proportional to size. Schools with ≥28 students per grade were defined as large, and those with <28 students were defined as small. The third sampling stage selected one or two classes within each grade of an SSU. All students in a selected class were eligible for ABES, unless they were unable to complete the questionnaire independently.

### Data Collection Procedures

The ABES study protocol was reviewed and approved by institutional review boards at CDC and ICF International, CDC’s survey contractor.* The survey was administered during January–June 2021; student participation was voluntary and anonymous. ABES was designed to be a self-administered questionnaire completed online during or outside of instructional time on any Internet-connected device. Most participating students (91%) completed ABES using a laptop or desktop computer; 9% of students used a phone or tablet computer.

### Data Processing and Response Rates

ABES data were cleaned and edited for inconsistencies. Values that were not plausible or logical were excluded from analysis (e.g., a student who answered that he had never smoked cigarettes but also answered that he had smoked cigarettes during the past 30 days). Before data editing, students in 128 schools submitted 7,998 ABES questionnaires (7,953 in English and 45 in Spanish). Among these 7,998 records, 293 were excluded because <20 questions had been answered, resulting in 7,705 questionnaires with valid data. The school response rate was 38%, the student response rate was 48%, and the overall response rate ([Student response rate] × [School response rate]) was 18%.

Race and ethnicity were ascertained using methods from the 2019 national YRBS (*6*); however, in this *MMWR Supplement,* the “other” race and ethnicity category was disaggregated to report the following racial categories (all non-Hispanic): American Indian or Alaska Native, Asian, Native Hawaiian or other Pacific Islander, and multiracial. The response options for sexual identity differed from those in the 2019 national YRBS (Table 1).

**TABLE 1:** Processing of sexual identity question — Adolescent Behaviors and Experiences Survey, United States, January–June 2021

Sexual identity question	Student response	Analytic coding
Which of the following best describes you?	Heterosexual (straight)	Heterosexual
	Gay or lesbian	Gay, lesbian, or bisexual
	Bisexual	
	I describe my sexual identity some other way.	Other or questioning
	I am not sure about my sexual identity (questioning).	
	I do not know what this question is asking.	Missing
	[Did not respond to the question]	

### Weighting

ABES data were weighted based on student sex and grade to account for school and student nonresponse and the oversampling of non-Hispanic Black students and Hispanic students. Weights were applied to all records and scaled so that weighted counts equaled the total sample size, and weighted student proportions in each grade matched national proportions. As a result, ABES weighted estimates are nationally representative of all students in grades 9–12 attending public and private schools in the United States.

### Analytic Methods

To account for the complex sampling design and weighting, ABES data were analyzed using SAS (version 9.4; SAS Institute) and SUDAAN (version 11.0.1 or 11.0.3; RTI International). The methods section of each report in this *MMWR Supplement* includes details on the analytical methods used in each analysis. For this overview report, a descriptive analysis of ABES student- and school-level data was conducted.

### Data Availability and Dissemination

ABES data and documentation describing the data are available to the public (https://www.cdc.gov/healthyyouth/data/abes.htm). Student survey and school characteristics data have been merged into one data set and are available in Access and ASCII formats. SAS and SPSS programs are provided for converting the ASCII data into SAS and SPSS data sets. No approval is needed to use the ABES data set. Data requests and other ABES-related questions can be sent to CDC by using the YRBS data request form (https://www.cdc.gov/healthyyouth/data/yrbs/contact.htm). 

## Results 

Among the high school students surveyed, 26.7% were in 9th grade, 25.5% in 10th grade, 24.3% in 11th grade, and 23.6% in 12th grade (Table 2). In addition, 50.4% of students were female and 49.8% were non-Hispanic White, 25.4% were Hispanic, and 12.9% were non-Hispanic Black. The remaining students reported other races (all non-Hispanic): American Indian or Alaska Native (0.7%), Asian (4.9%), multiracial (5.8%), and Native Hawaiian or other Pacific Islander (0.5%). A majority of students self-identified as heterosexual (77.5%); 13.2% self-identified as gay, lesbian, or bisexual; and 9.3% self-identified as other or questioning.

**TABLE 2:** Response rates and student characteristics — Adolescent Behaviors and Experiences Survey, United States, January–June 2021

Characteristic	No. (%)
Sample*	
Schools sampled	339
Students sampled	16,037
Participants*	
Schools	128
Students^†^	7,705
Response rates*	
Schools	(37.8)
Students	(48.0)
**Total**	**(18.1)**
**Sex^§,¶^**	
Female	3,999 (50.4)
Male	3,678 (49.6)
Race and ethnicity^§,^**	
American Indian or Alaska Native, non-Hispanic	83 (0.7)
Asian, non-Hispanic	350 (4.9)
Black, non-Hispanic	1,189 (12.9)
Hispanic or Latino	2,038 (25.4)
Multiracial, non-Hispanic	480 (5.8)
Native Hawaiian or other Pacific Islander, non-Hispanic	31 (0.5)
White, non-Hispanic	3,461 (49.8)
**Grade^§,††,§§^**	
9	2,144 (26.7)
10	1,949 (25.5)
11	1,858 (24.3)
12	1,731 (23.6)
**Sexual identity^§,¶¶^**	
Heterosexual	5,539 (77.5)
Gay, lesbian, or bisexual	977 (13.2)
Other or questioning	648 (9.3)
Language*^,^***	
English	7,953 (99.4)
Spanish	45 (0.6)

* Unweighted data.

^†^ Among 7,998 submitted questionnaires, 293 failed data validation and were excluded from analysis, resulting in 7,705 analyzable questionnaires.

^§^ Weighted data.

^¶^ 28 questionnaires were missing sex data.

** 73 questionnaires were missing race and ethnicity data.

^††^ 23 questionnaires had “ungraded” or “other” selected or missing.

^§§^ Percentages do not total 100% because of rounding.

^¶¶^ 541 questionnaires were missing sexual identity data.

*** Among 7,998 submitted questionnaires.

Of the 128 participating schools, 75.0% reported using a hybrid instructional mode, 21.9% used online instruction only, and 3.1% used in-person instruction only (Table 3). Approximately half of the schools (50.8%) had reduced class sizes. Most schools (94.5%) provided laptops or Chromebooks to students, and 60.3% provided Wi-Fi hotspots. 

**TABLE 3:** Participating school characteristics — Adolescent Behaviors and Experiences Survey, United States, January–June 2021

Characteristic	No. (%)
Total schools*	**128 (100)**
**Instructional model**	
In-person only	4 (3.1)
Virtual only	28 (21.9)
Hybrid^†^	96 (75.0)
**Reduced class size^§^**	
Yes	63 (50.8)
No	61 (49.2)
**School-provided equipment**	
Laptops or Chromebooks	
Yes	121 (94.5)
No	7 (5.5)
Tablet computers^¶^	
Yes	4 (3.3)
No	117 (96.7)
Wi-Fi hotspots**	
Yes	70 (60.3)
No	46 (39.7)

* Unweighted data.

^†^ Any combination of students attending in person and virtually.

^§^ Reduced class size information was missing for four schools.

^¶^ School-provided data on tablet computers were missing for seven schools.

** School-provided Wi-Fi hotspot data were missing for 12 schools.

## Discussion

CDC adapted successful school-based surveillance methods from the national YRBS to develop and launch ABES rapidly during the COVID-19 pandemic. In addition to using existing YRBS questions on the ABES survey, agencywide collaboration was used to develop new ABES questions, provide technical assistance on survey development and data collection, and conduct the analyses presented in this *MMWR Supplement*. ABES was the first national online survey of its kind available in both English and Spanish launched by CDC; online administration allowed students the flexibility to complete the survey during instructional time or on their own time. However, because schools faced numerous COVID-19–related challenges during the 2020–21 school year, certain districts and schools were reluctant to participate in ABES. For example, certain schools were concerned about loss of instructional time and the additional time required for school staff members. In some instances, contacting appropriate district- and school-level decision-makers to discuss participation was difficult because schools were minimally staffed or closed (ICF International, personal communication, 2021). Despite these challenges, ABES was still successfully administered, resulting in data that can be used to assess the impact of COVID-19 on various experiences and behaviors among high school students.

This is the first national school-based survey among high school students to assess a range of behaviors and experiences during the COVD-19 pandemic. A limited number of studies exist using other surveys among youths, but they have either had a narrower focus (e.g., focusing on one behavior such as substance use) (*8*) or are not nationally representative (*9*). With the varying school instructional models across the United States, obtaining national benchmark estimates for adolescent behaviors and experiences was imperative. Revised suppression criteria enabled the reporting of ABES findings for American Indian or Alaska Native, Asian, multiracial, and Native Hawaiian or other Pacific Islander students in this *MMWR Supplement* when data were available. This change allowed for a better understanding of behaviors and experiences among more specific groups of youths. Although the sampling methodology is the same for ABES and YRBS, findings should not be compared (i.e., prepandemic versus during pandemic) because administration modality and settings differed between surveys. YRBS uses a paper and pencil scannable survey that is administered in person in the schools; ABES used an online survey that students could complete during instructional time or on their own time, with access provided by teachers through schools.

## Limitations

The findings in this report are subject to at least five limitations. First, ABES data are representative of students enrolled in schools and are not representative of all U.S. adolescents, including those not enrolled in schools; an estimated 5% of adolescents aged 14–17 years were not enrolled in school in 2018 (*10*). Second, although ABES protocols were designed to protect students’ privacy, responses might have been visible to others during the survey, potentially leading to social desirability or response bias. Third, information on the instructional model being used by schools was collected at the time of school recruitment and might have changed during the study period. Therefore, interpreting findings among students who attended in-person, virtual, and hybrid school conditions is difficult, as is distinguishing in-person versus virtual attendance for the hybrid category. Fourth, because ABES was a one-time cross-sectional survey, causality or directionality of the findings cannot be determined, and changes in behaviors or experiences could not be assessed. Furthermore, the survey was not designed to allow comparisons by survey completion date; therefore, the results cannot be used to assess the impact of changes during the COVID-19 pandemic on responses. Finally, ABES had an 18% overall response rate; low response rates increase the potential for nonresponse bias. However, high response rates alone do not rule out nonresponse bias. Nonresponse bias analyses revealed that weighting adjustments minimized the potential for nonresponse bias because sample weights were adjusted to account for nonresponding schools (AM Roberts, R Iachan, L Harding, X Deng, ICF International, unpublished data, 2021).

## Conclusion

Collecting nationally representative data on behaviors and experiences among youths during the COVID-19 pandemic provides information on which behaviors are concerning and most prevalent during this time. ABES findings highlighted in this *MMWR Supplement* can help determine which risk behaviors are more prevalent among youths to assist parents, teachers, school administrators, community leaders, clinicians, and public health officials with addressing these issues. Additional surveillance is needed to monitor behaviors and experiences that are associated with the pandemic among youths across the United States.

## Footnotes

* 45 C.F.R. part 46; 21 C.F.R. part 56.
